# LncRNA HOXA11-AS promotes idiopathic pulmonary fibrosis progression via sponging miR-148a-3p and regulating SMAD2

**DOI:** 10.1186/s41065-026-00684-9

**Published:** 2026-05-14

**Authors:** Lidong Wang, Xing Ding, Ran Tang, Heping Jiang

**Affiliations:** Department of Respiratory and Critical Care Medicine, Changzhou Jintan First People’s Hospital, No. 500, Jintan Avenue, Jintan District, Changzhou, 213200 China

**Keywords:** HOXA11-AS, miR-148a-3p, SMAD2, Idiopathic pulmonary fibrosis

## Abstract

**Background:**

Idiopathic Pulmonary Fibrosis (IPF) is characterized primarily by progressive lung tissue scarring. This study aimed to explore the molecular mechanism of the HOXA11-AS/miR-148a-3p/SMAD2 axis in IPF.

**Methods:**

Sixty IPF patients and 70 healthy controls were recruited. mRNA expression was measured by RT-qPCR. An IPF model was established in A549 cells and MRC-5 cells using transforming growth factor-beta 1 (TGF-β1) induction. Cell proliferation, apoptosis, oxidative stress levels, and fibrosis markers were assessed using the cell counting kit-8 (CCK-8), flow cytometry, and enzyme-linked immunosorbent assay (ELISA), respectively. A dual-luciferase reporter assay was conducted to confirm the regulatory interaction.

**Results:**

In IPF patients, HOXA11-AS expression was significantly upregulated. Its expression was negatively correlated with lung function parameters as well as with the 6-minute walk test (6MWT) distance. Conversely, it was positively associated with fibrosis-related serum markers, such as hyaluronic acid (HA) and laminin (LN). These findings suggested that HOXA11-AS may serve as a promising diagnostic biomarker for IPF. Moreover, HOXA11-AS promoted the proliferation, suppressed apoptosis, enhanced oxidative stress by reducing superoxide dismutase (SOD) activity and increasing malondialdehyde (MDA) levels, and upregulated the key fibrosis markers expression, including alpha-smooth muscle actin (α-SMA), Collagen I, and Fibronectin. HOXA11-AS directly binds to miR-148a-3p, which in turn targets and regulates SMAD2. Notably, inhibition of miR-148a-3p reversed the suppressive effects of HOXA11-AS knockdown on TGF-β1-induced proliferation, oxidative stress, and fibrotic phenotypes in A549 cells, while restoring SMAD2 expression. Consistent results were observed in MRC-5 fibroblasts: HOXA11-AS knockdown significantly attenuated TGF-β1-induced proliferation, promoted apoptosis, and decreased the expression of α-SMA, Collagen I, and Fibronectin. These effects were partially reversed by miR-148a-3p inhibition, confirming the functional role of the HOXA11-AS/miR-148a-3p/SMAD2 axis in lung fibroblasts.

**Conclusions:**

HOXA11-AS promoted the IPF progression by upregulating SMAD2 expression through the sponging of miR-148a-3p.

**Supplementary Information:**

The online version contains supplementary material available at 10.1186/s41065-026-00684-9.

## Background

Idiopathic pulmonary fibrosis (IPF) is characterized primarily by progressive lung tissue scarring [1, 2]. This fibrotic process compromises gas exchange between the alveoli and pulmonary blood vessels [3], which leads to clinical manifestations such as dyspnea and dry cough. In advanced stages, patients may require supplemental oxygen, and the relentless progression of the disease can ultimately result in respiratory failure [4, 5]. Currently, the etiology of IPF remains elusive, its pathogenesis is not fully elucidated, and effective therapeutic options are limited [6, 7]. Therefore, further investigation into key molecular players involved in IPF pathogenesis and their underlying regulatory mechanisms is crucial for identifying novel diagnostic biomarkers.

Long non-coding RNAs (lncRNAs) are involved in the initiation and progression of various diseases through the regulation of microRNAs [8, 9]. HOXA11 antisense RNA (HOXA11-AS) is a lncRNA located on the antisense strand upstream of the homeobox A11 (HOXA11) [10], and its expression is significantly upregulated in multiple pathological conditions, including various cancers [11], keloids [12, 13], cardiac fibrosis [14], and hepatic fibrosis [15]. HOXA11-AS contributes to disease development by promoting fibroblast proliferation [16] and triggering oxidative stress and inflammatory responses [17]. However, the HOXA11-AS expression profile in IPF and its potential regulatory mechanisms remain largely unexplored. Moreover, microRNA-148a-3p (miR-148a-3p) can inhibit collagen synthesis and secretion in fibroblasts [18] or alleviate fibrotic progression by inhibiting the β-catenin signaling pathway [19]. TGF-β1/SMAD pathway is a key signaling cascade involved in the regulation of pulmonary fibrosis [20, 21]. Among its components, SMAD2 functions as a core effector molecule [22], and upon activation, translocates into the nucleus to regulate the expression of fibrosis-related genes, such as alpha-smooth muscle actin (α-SMA) and Collagen [23]. SMAD2 is highly expressed in IPF patients [24], further promoting the fibrotic process, with its aberrant overexpression being closely associated with disease severity [25]. Bioinformatics predictions indicate HOXA11-AS targeted miR-148a-3p, as well as miR-148a-3p targeted SMAD2. These findings suggest that the three molecules may form a competing endogenous RNA (ceRNA) regulatory axis, thereby participating in the initiation and progression of IPF.

Through clinical sample analysis and in vitro cell experiments, this study systematically evaluated the HOXA11-AS expression in IPF, assessed its potential diagnostic significance, elucidated the regulatory mechanism of the HOXA11-AS/miR-148a-3p/SMAD2 axis, and further investigated the function of this regulatory pathway in oxidative stress responses and fibrotic progression in IPF.

## Methods

### Participants

Sixty IPF patients and 70 healthy controls were recruited from Changzhou Jintan First People’s Hospital. The IPF diagnosis was established pursuant to the 2022 Diagnostic Guidelines for Idiopathic Pulmonary Fibrosis issued by the American Thoracic Society/European Respiratory Society/Japanese Respiratory Society/Latin American Thoracic Association (ATS/ERS/JRS/ALAT) [26]. The exclusion criteria were as follows: presence of acute exacerbation of IPF, recent respiratory tract infection within 4 weeks before screening, comorbid conditions such as liver cirrhosis, renal failure, malignancies, autoimmune diseases, diabetes mellitus, severe osteoporosis, or tuberculosis, prior lung transplantation, and pregnancy or lactation.

All participants fasted overnight and had 5 mL of peripheral venous blood collected. Following serum separation, the samples were stored in a -80 °C freezer. Pulmonary function was assessed using the MasterScreen PFT pulmonary function analyzer (Jaeger, Germany). The measured parameters included forced vital capacity (FVC) and diffusing capacity for carbon monoxide (DLCO). Exercise capacity was evaluated using the 6-minute walk test (6MWT). If participants experienced symptoms such as dizziness or dyspnea during the test, the procedure was to be terminated immediately.

This study was performed in line with the principles of the Declaration of Helsinki. Approval was granted by the Ethics Committee of Changzhou Jintan First People’s Hospital. All participants provided written informed consent.

### Cell culture and transfection

The human alveolar epithelial cell line A549 (ATCC) and the human lung fibroblast MRC-5 cell line (BeNa Culture) were cultured in Dulbecco’s Modified Eagle’s Medium (DMEM, Gibco), supplemented with 10% fetal bovine serum (FBS) (Thermo Fisher Scientific), penicillin, and streptomycin, and maintained in a humidified incubator at 37 °C with 5% CO₂. When the cells reached approximately 80% confluency, they were treated with 10 ng/mL TGF-β1 (R&D Systems, Inc.) [27, 28] for 48 h to induce an IPF cell model. A549 cells were selected for this study due to their well-characterized, homogeneous epithelial phenotype, high reproducibility, and widespread use as a surrogate model for alveolar epithelial type II cells in mechanistic studies of pulmonary fibrosis, facilitating the investigation of cell-intrinsic signaling pathways under controlled conditions [29, 30]. A549 cells have been widely validated as a reliable cell model for investigating IPF in numerous studies [31, 32].

Small interfering RNA targeting HOXA11-AS (si-HOXA11-AS), miRNA mimics/inhibitors, and their corresponding negative controls (NC) were synthesized by Guangzhou RiboBio Co., Ltd. Transfection was carried out using Lipofectamine 2000 (Thermo Fisher Scientific) in accordance with the experimental group designations. After 48 h of transfection, cells were harvested for subsequent experiments.

### RT-qPCR analysis of gene expression

Total RNA was extracted from serum, A549 cells and MRC-5 cells using the TRIzol method (Invitrogen), and complementary DNA (cDNA) was synthesized through reverse transcription with the PrimeScript RT Kit (Takara). RT-qPCR was subsequently carried out using SYBR Green Master Mix (Applied Biosystems). GAPDH and U6 were used as internal references.

### Proliferation

After transfection, the A549 cells and MRC-5 cells were seeded into a 96-well plate at a density of 1 × 10⁴ cells per well. At predetermined time points, 10 µL of CCK-8 reagent (Cell Counting Kit-8) was added to each well. Following incubation at 37 °C for 2 h, the OD at 450 nm was measured to assess cell proliferation capacity.

### Apoptosis

After transfection, the A549 cells and MRC-5 cells were harvested and stained using an Annexin V-FITC/PI Apoptosis Detection Kit (BD Biosciences), followed by a 15-minute incubation in the dark. Cell apoptosis was subsequently analyzed using a flow cytometer (FACSCanto II flow cytometer, BD Biosciences) with FlowJo 7.6 software for data processing.

### Oxidative stress indicators

Malondialdehyde (MDA) was quantified using an MDA Assay Kit (S0131S, Beyotime), while superoxide dismutase (SOD) activity was determined using an SOD Assay Kit (Jiancheng Bioengineering Institute).

### Enzyme-linked immunosorbent assay (ELISA)

ELISA kits were employed to quantify the serum levels of hyaluronic acid (HA, Solarbio) and laminin (LN, Jianglai Bio). Additionally, the α-SMA levels (Lianshuo Biological Technology), Collagen I, and fibronectin (Thermo Fisher Scientific) in A549 cells and MRC-5 cells were assessed using similar kits. The microplates included in the kits were pre-coated with antibodies specific to each target molecule. Following this, samples, standards, or control solutions were added to designated wells to facilitate antigen-antibody binding. Subsequently, the corresponding secondary antibody and substrate solution were introduced, and the resulting enzymatic reaction was quantified by measuring the optical signal.

### Dual luciferase assay

The wild-type (WT) and mutant (MUT) sequences of HOXA11-AS and the 3’UTR of SMAD2 were synthesized and cloned into the pGL3 luciferase reporter vector (Promega) by Guangzhou RiboBio Co., Ltd. A549 cells were seeded into 24-well plates and transfected when the cell confluency reached approximately 80%. The reporter gene vectors were co-transfected with miR-148a-3p mimics or inhibitors and their respective negative controls. After 48 h, luciferase activity was measured using the Dual-Luciferase Reporter Assay System (Promega).

### Subcellular fraction assay

The subcellular localization of HOXA11-AS was determined using the PARISTM Kit (Invitrogen). MRC-5 cells were suspended in cytoplasmic lysis buffer, and the cytoplasmic supernatant was collected by centrifugation. The remaining pellet was resuspended in nuclear lysis buffer at 4 °C for 1 h, followed by centrifugation for 10 min. RNA from cytoplasmic and nuclear extracts was purified using TRIzol. The expression levels of GAPDH (cytoplasmic marker), U6 (nuclear marker), and HOXA11-AS in the nucleus and cytoplasm were quantified by RT‑qPCR.

### Statistical analysis

Statistical analysis was conducted using SPSS 26.0 (IBM) and GraphPad Prism 9.0. The normality of data distribution was assessed using Shapiro-Wilk test. For comparisons between two groups, independent samples t-test (for normally distributed data) or Mann-Whitney U test (for non-normally distributed data) was applied. For comparisons among multiple groups, Kruskal-Wallis test followed by Dunn’s post-hoc test with Bonferroni correction was used. All in vitro experiments were performed with at least three independent biological replicates, and each experiment included three technical replicates. Pearson correlation coefficients were calculated to assess the relationships between variables, while the diagnostic performance was evaluated using receiver operating characteristic (ROC) curves. A *P* < 0.05 was considered statistically significant.

## Results

### Clinical features

A comparative analysis of the clinical characteristics between 70 healthy controls and 60 IPF patients revealed no statistically significant differences in age, gender, smoking status, or body mass index (BMI) between the two groups. However, lung function parameters (DLCO and FVC), exercise capacity (6MWT), and extracellular matrix biomarkers associated with pulmonary fibrosis—specifically HA and LN-showed significant differences between the IPF and control (Table 1).

**Table 1:** Clinical characteristics in IPF patients and health controls

Characteristics	Control (*n* = 70)	IPF (*n* = 60)	*P*-value
Age (years)	66.36 ± 6.37	67.33 ± 6.15	0.378
Gender (male/female)	41/29	42/18	0.176
Smoking (yes/no)	22/48	26/34	0.161
BMI (kg/m^2^)	23.13 ± 2.15	23.78 ± 2.26	0.096
DLCO (%)	86.69 ± 5.94	62.36 ± 8.88	< 0.001
FVC (%)	86.93 ± 5.97	63.56 ± 10.00	< 0.001
6MWT (meters)	446.84 ± 72.90	301.32 ± 124.46	< 0.001
HA (ng/ml)	49.17 ± 16.64	246.63 ± 65.90	< 0.001
LN (ng/ml)	20.16 ± 3.96	64.35 ± 14.66	< 0.001

Continuous variables were presented as mean ± standard deviation (SD) and were compared between groups using the independent samples t-test. Categorical variables were expressed as N/ N and were analyzed using the chi-square test

*IFP* idiopathic pulmonary fibrosis, *BMI* body mass index, *DLCO* capacity for carbon monoxide, *FVC* forced vital capacity, *6MWT* six-minute walk test, *HA* hyaluronic acid, *LN* laminin

A *P*-value < 0.05 was considered statistically significant

### Dysregulation of HOXA11-AS in IPF

**Fig. 1:**
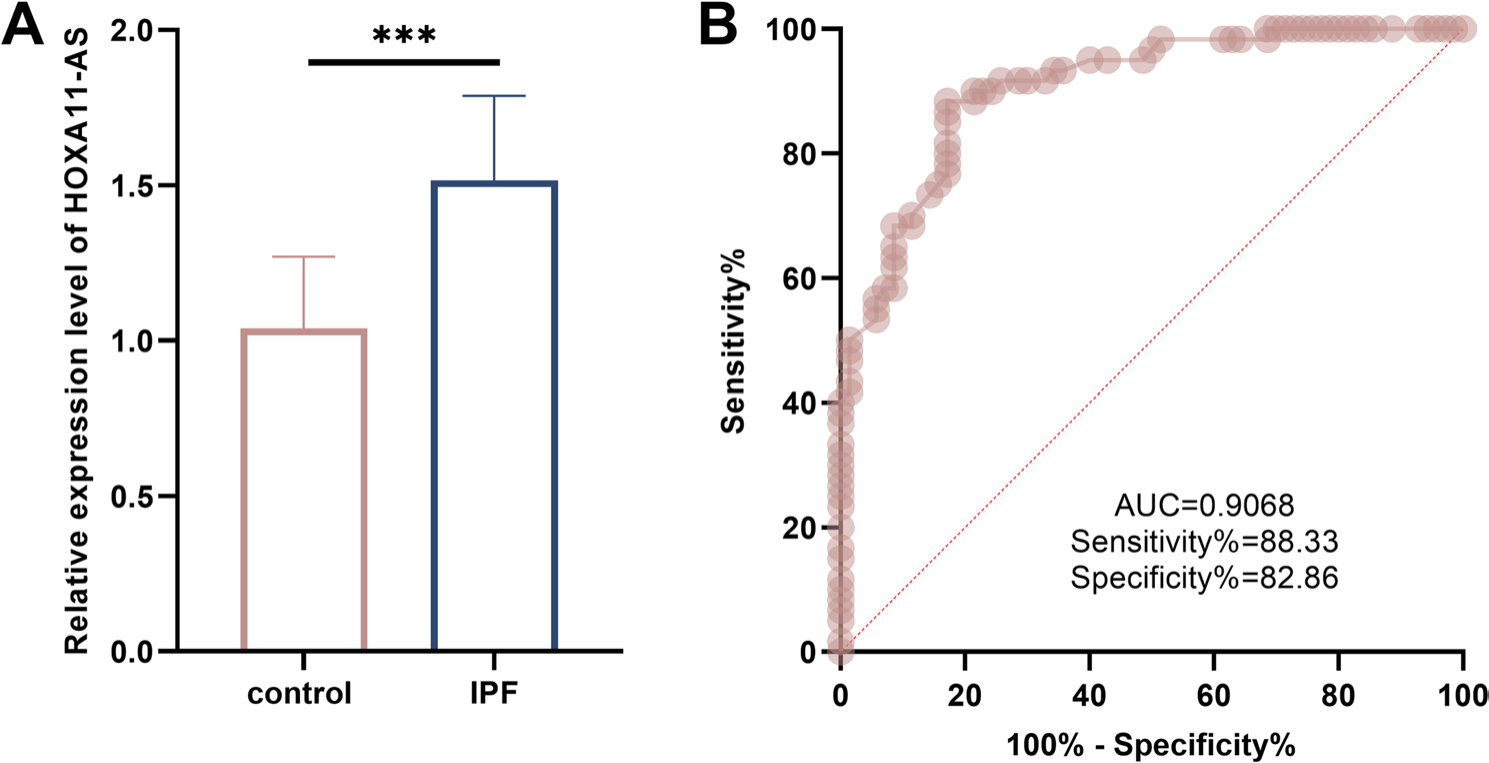
Elucidation of the role of HOXA11-AS in IPF. **A** HOXA11-AS was significantly upregulated in IPF patients. **B** HOXA11-AS may serve as a potential diagnostic biomarker for IPF. *** means *P* < 0.001. Data were presented as mean ± SD. Statistical comparisons were performed using independent Student’s t-test for panel A and ROC curve analysis for panel B

HOXA11-AS was highly expressed in IPF (Fig. 1A), suggesting its potential as a diagnostic biomarker for IPF. The area under the curve (AUC) was 0.9068, indicating a sensitivity of 88.33% and a specificity of 82.86% (Fig. 1B).

### Association between HOXA11-AS expression and fibrosis-related biomarkers in IPF

HOXA11-AS expression was significantly and negatively correlated with lung function parameters (DLCO: *r* = -0.692, FVC: *r* = -0.622). It also exhibited a strong negative association with 6MWT (*r* = -0.786). Furthermore, HOXA11-AS demonstrated a significant positive correlation with extracellular matrix markers associated with pulmonary fibrosis (HA: *r* = 0.703, LN: *r* = 0.807) (Table 2).

**Table 2:** Correlation analysis of HOXA11-AS expression with fibrosis indicators of IPF

Items	*r*	*P*-value
DLCO (%)	-0.692	< 0.001
FVC (%)	-0.622	< 0.001
6MWT (meters)	-0.786	< 0.001
HA (ng/ml)	0.703	< 0.001
LN (ng/ml)	0.807	< 0.001

Pearson correlation coefficient analysis was performed to evaluate the linear relationships between HOXA11-AS expression and clinical parameters (DLCO, FVC, 6MWT, HA, and LN)

*IFP* idiopathic pulmonary fibrosis, *DLCO* capacity for carbon monoxide, *FVC* forced vital capacity, *6MWT* six-minute walk test, *HA* hyaluronic acid, *LN* laminin

The correlation strength was assessed by the r value, and a two-tailed *P* < 0.05 was considered statistically significant

### Effect of HOXA11-AS on cell function under the induction of TGF-β1

**Fig. 2:**
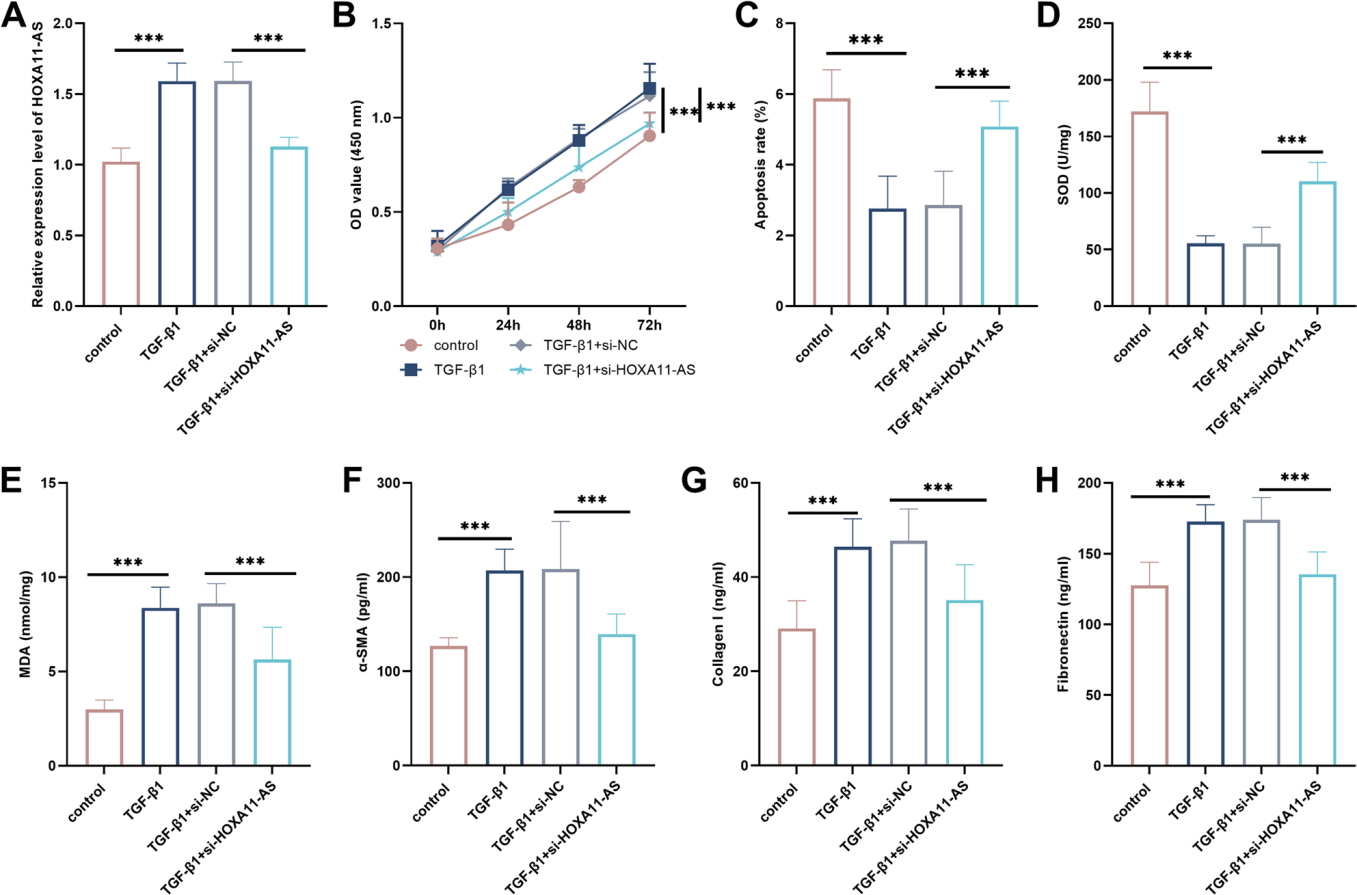
Effects of HOXA11-AS on cellular functions under TGF-β1 induction. **A** TGF-β1 stimulation markedly increases HOXA11-AS expression, which can be effectively reduced by si-HOXA11-AS transfection. **B** HOXA11-AS enhances TGF-β1-induced cell proliferation. **C** HOXA11-AS suppresses TGF-β1-induced apoptosis. **D** HOXA11-AS impairs cellular antioxidant capacity. **E** HOXA11-AS exacerbates oxidative stress-induced damage. **F** HOXA11-AS promotes the expression of α-SMA. **G** HOXA11-AS enhances the expression of Collagen I. **H** HOXA11-AS upregulates Fibronectin expression. *** means *P* < 0.001. Data were presented as mean ± SD. Statistical comparisons among multiple groups were performed using Kruskal-Wallis test followed by Dunn’s post-hoc test with Bonferroni correction

The subcellular distribution of HOXA11-AS in MRC-5 cells was detected by cell subcellular fractionation technology in combination with RT-qPCR. The results showed that HOXA11-AS was detected in both the cytoplasm and the nucleus of the cells, with the content in the cytoplasm being particularly significant (Supplementary Fig. 1). After stimulation with TGF-β1, HOXA11-AS expression was markedly upregulated, whereas transfection with si-HOXA11-AS effectively suppressed its expression (Fig. 2A). In the TGF-β1-induced cell model, HOXA11-AS enhanced cell proliferation (Fig. 2B), attenuated cell apoptosis (Fig. 2C), and diminished antioxidant capacity, as indicated by decreased SOD levels (Fig. 2D) and elevated MDA levels (Fig. 2E), thereby aggravating oxidative damage. Furthermore, HOXA11-AS upregulated the expression of fibrosis-related markers, including α-SMA (Fig. 2F), Collagen I (Fig. 2G), and fibronectin (Fig. 2H), thereby contributing to the progression of fibrosis.

### The targeting interaction between HOXA11-AS and miR-148a-3p

**Fig. 3:**
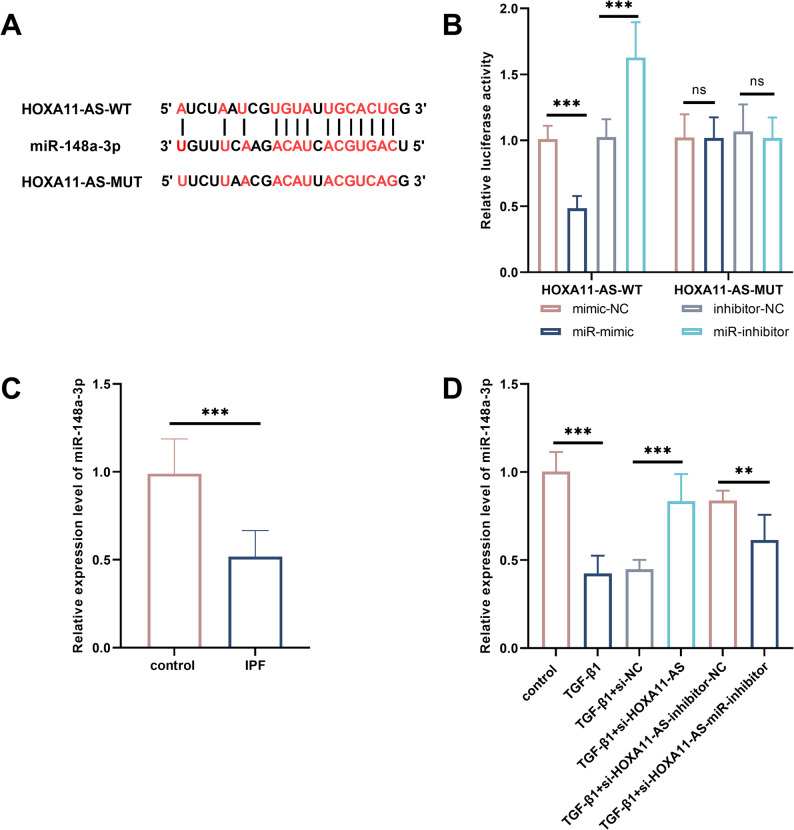
The direct interaction between HOXA11-AS and miR-148a-3p. **A** Bioinformatic analysis predicts potential binding sites between HOXA11-AS and miR-148a-3p. **B** Dual-luciferase reporter assays confirm the direct binding between HOXA11-AS and miR-148a-3p. **C** miR-148a-3p expression was significantly downregulated in IPF patients compared to controls. **D** Knockdown of HOXA11-AS restores miR-148a-3p expression. ns indicates no statistical significance. ** means *P* < 0.01, *** means *P* < 0.001. Data were presented as mean ± SD. Statistical comparisons were performed using independent Student’s t-test for panel **C**. For panels **B **and **D**, comparisons among multiple groups were performed using Kruskal-Wallis test followed by Dunn’s post-hoc test with Bonferroni correction

HOXA11-AS could directly bind to miR-148a-3p. Following transfection with the miR-148a-3p mimic, luciferase activity in the HOXA11-AS-WT group was significantly reduced. Conversely, transfection with the miR-148a-3p inhibitor led to a significant increase in luciferase activity in the same group. In contrast, no notable changes were observed in the HOXA11-AS-MUT group (Figs. 3A-B). Furthermore, miR-148a-3p expression was markedly decreased in IPF patients (Fig. 3C). Upon TGF-β1 stimulation, suppression of HOXA11-AS expression resulted in a significant upregulation of miR-148a-3p levels. These findings suggested that HOXA11-AS functions as a ceRNA and contributed to the regulation of IPF pathogenesis by sequestering miR-148a-3p (Fig. 3D).

### ***HOXA11-AS/miR-148a-3p axis in the TGF-β1-induced A549 model***

**Fig. 4:**
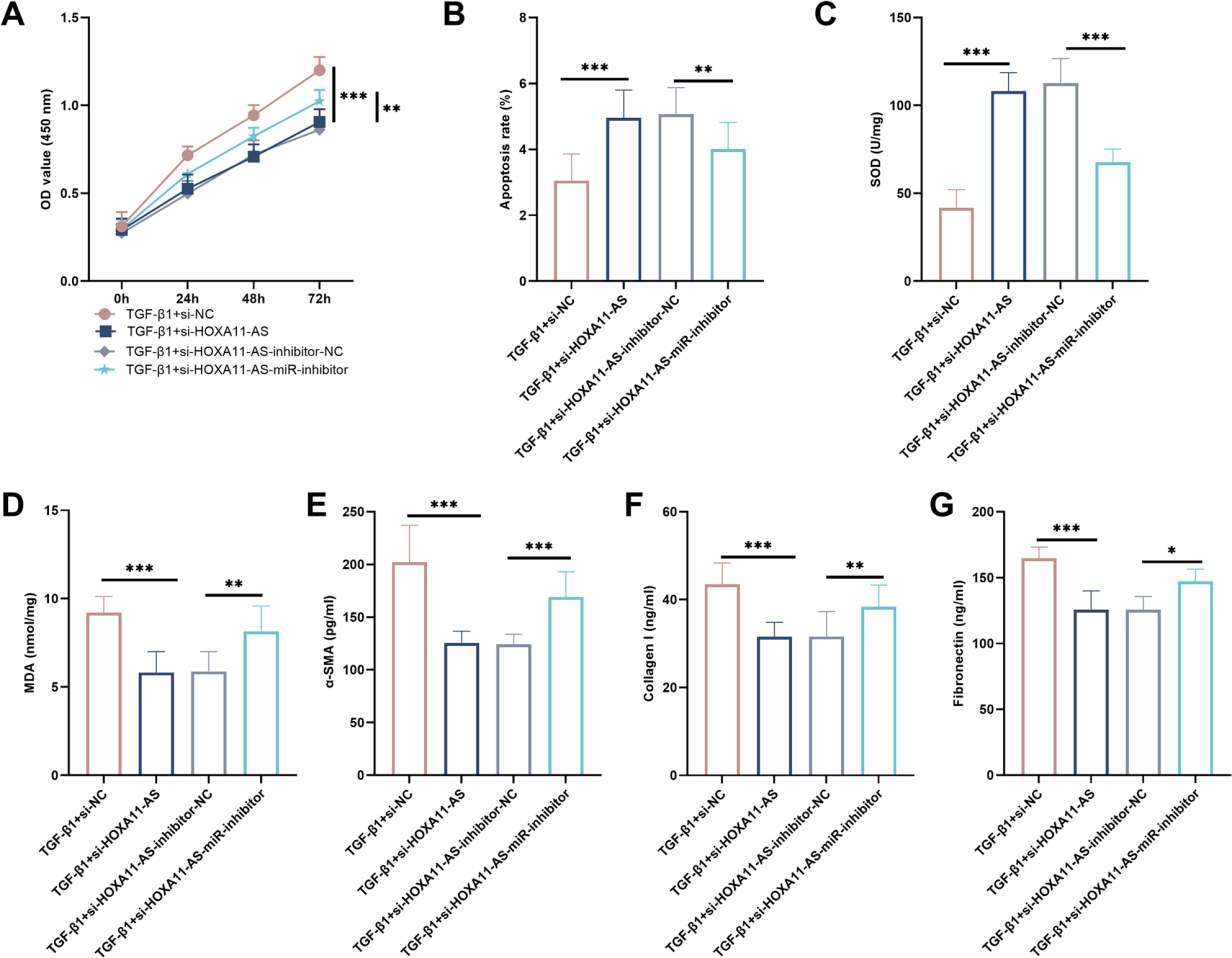
Regulatory effects of the HOXA11-AS/miR-148a-3p axis on cellular functions in the TGF-β1-induced model. **A** miR-148a-3p is involved in HOXA11-AS-mediated regulation of cell proliferation. **B** miR-148a-3p mediates HOXA11-AS-regulated apoptosis. **C** miR-148a-3p participates in HOXA11-AS-regulated SOD levels. **D** miR-148a-3p mediates HOXA11-AS-regulated MDA levels. **E** miR-148a-3p is involved in HOXA11-AS-regulated α-SMA expression. **F** miR-148a-3p mediates HOXA11-AS-regulated Collagen I expression. **G** miR-148a-3p participates in HOXA11-AS-regulated Fibronectin expression. * means *P* < 0.05, ** means *P* < 0.01, *** means *P* < 0.001. Data were presented as mean ± SD. Statistical comparisons among multiple groups were performed using Kruskal-Wallis test followed by Dunn’s post-hoc test with Bonferroni correction

In the TGF-β1-induced cell model, HOXA11-AS influenced cellular functions through the regulation of miR-148a-3p. Inhibition of miR-148a-3p partially blocked the biological cascade initiated by si-HOXA11-AS—it not only reversed the inhibitory effect of si-HOXA11-AS on cell proliferation (Fig. 4A) and its pro-apoptotic effect (Fig. 4B), but also mitigated the si-HOXA11-AS-induced enhancement of cellular antioxidant capacity, as reflected by changes in SOD and MDA levels (Fig. 4C-D). Furthermore, miR-148a-3p inhibition significantly suppressed the downregulation of fibrosis markers triggered by si-HOXA11-AS, including Collagen I and fibronectin (Fig. 4F-G).

### miR-148a-3p targeted SMAD2

**Fig. 5:**
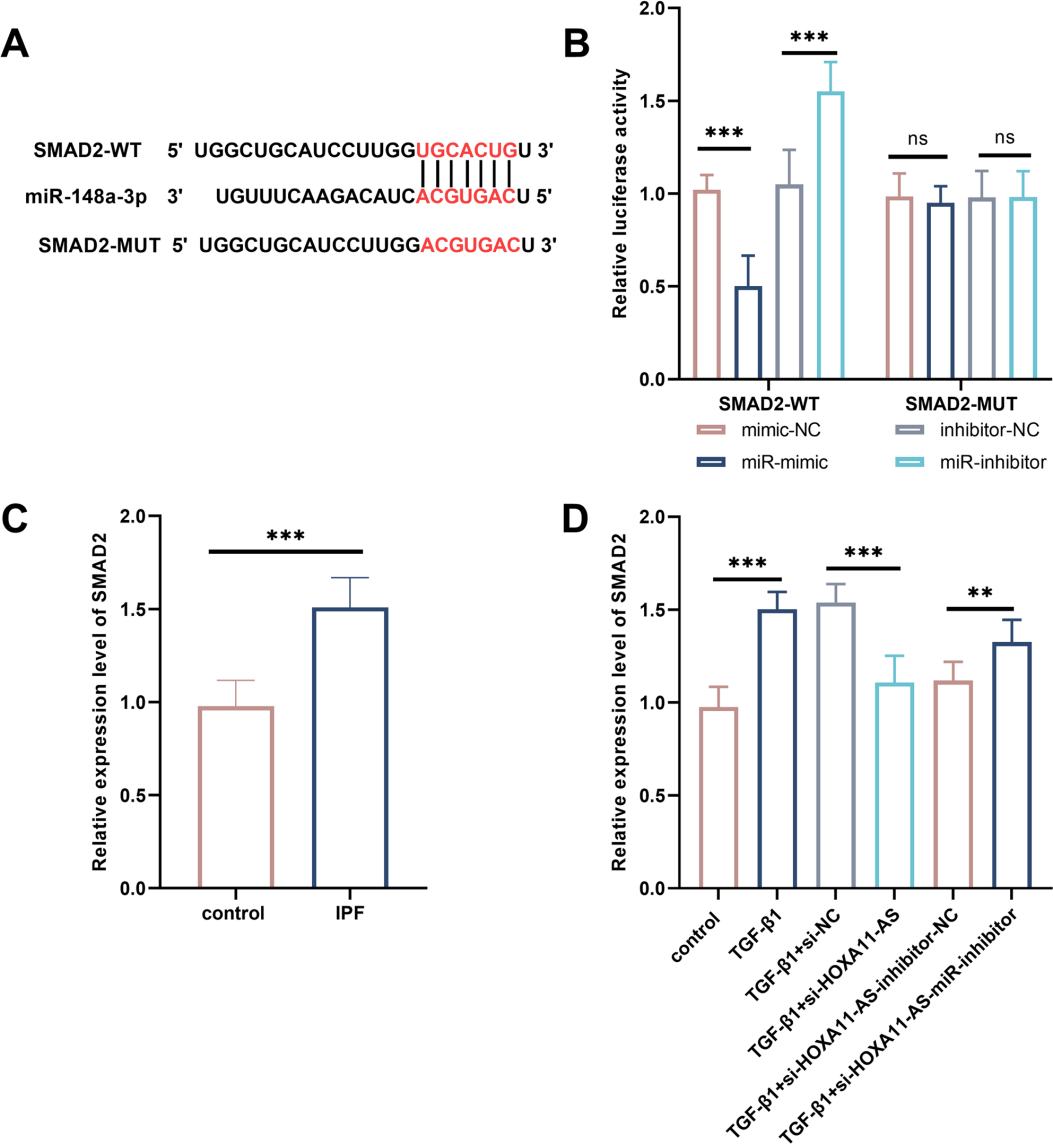
Targeting interaction between miR-148a-3p and SMAD2. **A** Putative binding sites between miR-148a-3p and SMAD2 were predicted. **B** Dual-luciferase reporter assays confirm the direct binding between miR-148a-3p and SMAD2. **C** SMAD2 is significantly overexpressed in IPF patients. **D** HOXA11-AS functions as a molecular sponge for miR-148a-3p, thereby alleviating its inhibitory effect on SMAD2 and promoting SMAD2 upregulation, which contributes to TGF-β1-induced fibrosis. ns indicates no statistical significance. ns means no statistical significance, ** means *P* < 0.01, *** means *P* < 0.001. Data were presented as mean ± SD. Statistical comparisons were performed using independent Student’s t-test for panel **C**. For panels **B** and **D**, comparisons among multiple groups were performed using Kruskal-Wallis test followed by Dunn’s post-hoc test with Bonferroni correction

miR-148a-3p could directly bind to SMAD2 (Figs. 5A-B). Furthermore, SMAD2 expression was markedly upregulated in IPF patients (Fig. 5C). HOXA11-AS functions as a competitive sponge for miR-148a-3p, thereby alleviating the inhibitory effect of miR-148a-3p on SMAD2. This results in further upregulation of SMAD2 expression and contributes to the TGF-β1-induced fibrotic process (Fig. 5D).

### ***HOXA11-AS/miR-148a-3p axis in the TGF-β1-induced MRC-5 model***

**Fig. 6:**
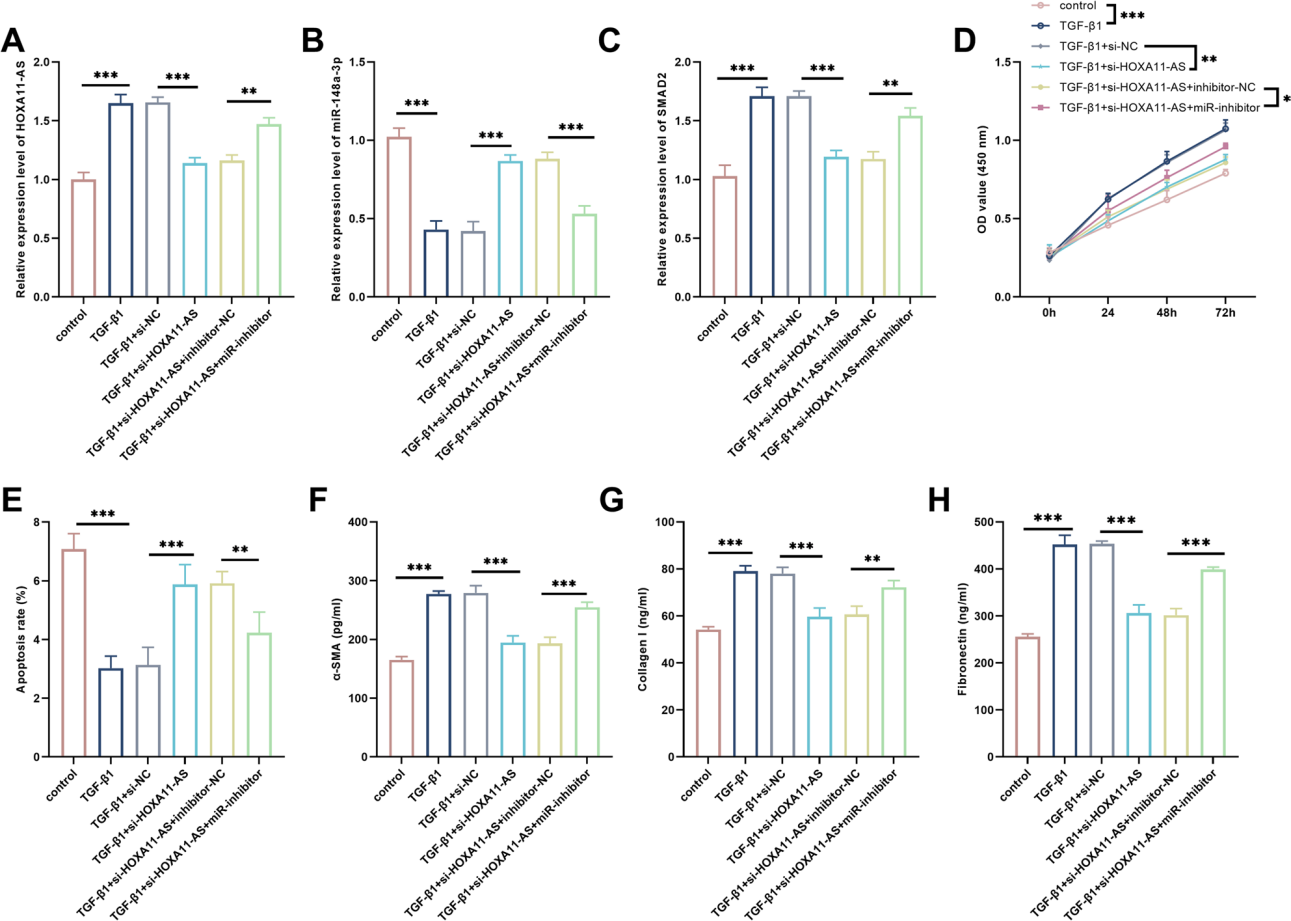
HOXA11-AS/miR-148a-3p/SMAD2 axis regulated fibrotic phenotypes in TGF-β1-induced MRC-5 fibroblasts. **A-C** Relative expression of HOXA11-AS (**A**), miR-148a-3p (**B**), and SMAD2 (**C**) in MRC-5 cells under indicated treatments. **D** Cell proliferation assessed by CCK-8 assay. **E** Apoptosis rate determined by flow cytometry. **F-H** Expression of fibrosis markers: α-SMA (**F**), Collagen I (**G**), and Fibronectin (**H**) measured by ELISA. *P < 0.05, **P < 0.01, ***P < 0.001. Data were presented as mean ± SD. Statistical comparisons among multiple groups were performed using Kruskal-Wallis test followed by Dunn’s post-hoc test with Bonferroni correction

To further validate the functional role of the HOXA11-AS/miR-148a-3p/SMAD2 axis in lung fibroblasts, we performed similar experiments in MRC-5 cells. As shown in Fig. 6, TGF-β1 stimulation significantly upregulated HOXA11-AS expression (Fig. 6A), downregulated miR-148a-3p (Fig. 6B), and increased SMAD2 expression (Fig. 6C) in MRC-5 cells. Knockdown of HOXA11-AS using si-HOXA11-AS effectively reversed these changes, while co-transfection with miR-148a-3p inhibitor partially restored SMAD2 expression (Fig. 6C). Functional assays revealed that TGF-β1 significantly promoted MRC-5 cell proliferation (Fig. 6D), inhibited apoptosis (Fig. 6E), and upregulated the expression of fibrosis markers α-SMA (Fig. 6F), Collagen I (Fig. 6G), and Fibronectin (Fig. 6H). HOXA11-AS knockdown significantly attenuated these TGF-β1-induced effects, and miR-148a-3p inhibition partially reversed the protective effects of si-HOXA11-AS. These results demonstrated that the HOXA11-AS/miR-148a-3p/SMAD2 axis also played a critical role in regulating fibroblast activation and fibrotic responses in IPF.

## Discussion

The early diagnosis of IPF primarily depends on high-resolution computed tomography (HRCT) and lung biopsy [33]. However, HRCT has limited sensitivity for detecting early-stage lesions, and lung biopsy, being an invasive procedure, presents certain clinical limitations [34]. Therefore, identifying non-invasive molecular biomarkers with high specificity is crucial for enabling early intervention in IPF. HOXA11-AS is involved in the regulation of fibrotic processes [35, 36], yet its specific regulatory mechanism in IPF remains poorly understood. This study was the first to systematically investigate the role and underlying molecular mechanism of HOXA11-AS in IPF. We found that HOXA11-AS was significantly upregulated in IPF patients and exhibited potential as a diagnostic biomarker. Mechanistically, HOXA11-AS functions as a ceRNA by sponging miR-148a-3p, leading to the upregulation of its target gene SMAD2 and thereby promoting IPF progression.

HOXA11-AS was significantly elevated in IPF and suggested potential diagnostic utility. Moreover, HOXA11-AS expression was significantly and negatively correlated with lung function parameters (DLCO, FVC), while showing a positive correlation with biomarkers associated with extracellular matrix (ECM) deposition (HA, LN). These findings indicated that HOXA11-AS may not only serve as a potential diagnostic biomarker for IPF but also reflect disease severity. This observation aligned with the reported roles of HOXA11-AS in other fibrotic conditions: in keloids, elevated HOXA11-AS expression is associated with increased scar formation [12, 17], and in cardiac fibrosis, it promotes the fibrotic transformation of cardiac fibroblasts [14]. Collectively, these findings suggested that HOXA11-AS may function as a common regulatory molecule across multiple fibrotic diseases.

Oxidative stress serves as a key factor in alveolar epithelial injury and the initiation of fibrosis in IPF [37, 38]. The underlying mechanisms involve the disruption of the alveolar epithelial barrier function and the activation of the TGF-β1 signaling pathway, which in turn promotes fibroblast activation and ECM synthesis [39, 40]. This study was the first to demonstrate that HOXA11-AS expression was significantly elevated in both IPF clinical samples and TGF-β1-induced cell models. Silencing HOXA11-AS significantly inhibited A549 cell proliferation, enhanced apoptosis, alleviated oxidative stress (increased SOD and reduced MDA), and downregulated the expression of fibrosis-related proteins such as α-SMA, Collagen I, and fibronectin. Consistent results were obtained in MRC-5 fibroblasts, where HOXA11-AS knockdown attenuated TGF-β1-induced proliferation, apoptosis resistance, and ECM production, indicating that HOXA11-AS exerted pro-fibrotic effects in both epithelial cells and fibroblasts. These findings suggested that HOXA11-AS contributed to the transition of alveolar epithelial cells into a fibrotic phenotype by exacerbating oxidative stress. This mechanism was consistent with the previous studies: HOXA11-AS is involved in UVB-induced oxidative damage [41] and exacerbates oxidative stress and inflammatory responses by epigenetically suppressing the Nrf2 antioxidant pathway, thereby promoting keloid development [17]. This study further confirmed the regulatory function of HOXA11-AS in oxidative stress during pulmonary fibrosis, indicating that oxidative stress may represent a common mechanism through which HOXA11-AS participates in various fibrotic diseases.

The ceRNA mechanism represents a crucial regulatory pathway through which lncRNAs modulate gene expression [42, 43]. In this study, a substantial proportion of HOXA11-AS was also distributed in the cytoplasm, which is critical for its role as a ceRNA, enabling it to sequester target miRNAs. Notably, it was confirmed that HOXA11-AS directly binds to miR-148a-3p, which in turn targets and regulates SMAD2. In IPF patients, HOXA11-AS expression was elevated, miR-148a-3p expression was reduced, and SMAD2 was upregulated. Silencing HOXA11-AS significantly increased miR-148a-3p expression while decreasing SMAD2 expression. Conversely, inhibition of miR-148a-3p restored SMAD2 expression and counteracted the anti-fibrotic effects induced by HOXA11-AS knockdown. These findings were recapitulated in MRC-5 fibroblasts, confirming that the HOXA11-AS/miR-148a-3p/SMAD2 axis operates in both alveolar epithelial cells and lung fibroblasts, the two major cell types involved in IPF pathogenesis. These findings elucidated the regulatory axis of HOXA11-AS/miR-148a-3p/SMAD2. The TGF-β1/SMAD signaling pathway contributes significantly to the pulmonary fibrosis pathogenesis [44]. Upon activation, SMAD2, as a key transcription factor, translocates into the nucleus to regulate the expression of fibrosis-related genes [45]. Previous studies have shown that SMAD2 is significantly upregulated in IPF patients, and its suppression can ameliorate fibrotic progression [24, 25].

Meanwhile, miR-148a-3p is downregulated in pulmonary fibrosis and exerts anti-fibrotic effects by targeting genes such as Hsp90b1 and β-catenin [18, 19]. This study is the first to establish a functional link among these three molecules, demonstrating that HOXA11-AS activates the SMAD2-mediated fibrotic pathway by acting as a molecular sponge for miR-148a-3p. This finding clarified the molecular mechanism by which HOXA11-AS promoted IPF progression and provided novel insights into the upstream regulatory mechanisms of the TGF-β1/SMAD signaling pathway.

Clinically, HOXA11-AS expression may be measured and utilized as a non-invasive auxiliary diagnostic biomarker for identifying early suspected cases and complementing the diagnosis of patients who are unable to undergo a lung biopsy. Furthermore, it may serve as an indicator for assessing disease severity and prognosis, aiding in patient stratification and prognostic monitoring during treatment, thereby guiding adjustments in follow-up frequency and therapeutic strategies. However, this study has several limitations. First, the findings were based exclusively on in vitro cell models (A549 and MRC-5), and the absence of in vivo validation—such as in a bleomycin-induced pulmonary fibrosis mouse model—limited the demonstration of the HOXA11-AS/miR-148a-3p/SMAD2 axis in a complex biological environment. Second, the use of A549 cells, a lung adenocarcinoma cell line, may not fully recapitulate the pathophysiology of primary alveolar epithelial cells, although we complemented the experiments with MRC-5 fibroblasts to validate the regulatory axis across different cell types. Third, the mechanistic exploration primarily focused on the ceRNA “sponging” model, and the downstream transcriptional regulation of fibrotic genes, as well as SMAD2 nuclear translocation dynamics, remain to be further elucidated. Fourth, the diagnostic potential of HOXA11-AS, while promising (AUC = 0.9068), was evaluated in a relatively small, single-center cohort without inclusion of other interstitial lung diseases as controls, limiting the assessment of its specificity for IPF. Finally, although we performed subcellular fractionation to confirm the cytoplasmic localization of HOXA11-AS, RNA in situ hybridization on IPF lung tissues was still needed to determine its cell type-specific distribution at the lesion site. To address these limitations, future studies will establish HOXA11-AS knockout or knockdown mouse models to evaluate its in vivo effects on lung tissue fibrosis; incorporate primary human alveolar epithelial cells and utilize chromatin immunoprecipitation (ChIP), immunofluorescence co-localization, and nuclear-cytoplasmic fractionation to dissect the precise transcriptional regulation and SMAD2 nuclear dynamics; conduct multi-center, large-scale cohort studies including other interstitial lung diseases to validate the diagnostic specificity of HOXA11-AS; and perform RNA in situ hybridization on IPF lung tissue sections to visualize its cell type-specific distribution. These investigations will provide a more comprehensive understanding of the HOXA11-AS-mediated fibrotic progression and strengthen the translational relevance of our findings.

## Conclusion

HOXA11-AS promoted the IPF progression by upregulating SMAD2 expression through the sponging of miR-148a-3p. This mechanism may offer a novel theoretical basis for understanding IPF pathogenesis. 

## Supplementary Information

Supplementary Material 1: Figure 1. Subcellular localization of HOXA11-AS in MRC-5 cells. Subcellular fractionation was performed in MRC-5 cells, followed by RT-qPCR analysis of HOXA11-AS expression in the cytoplasmic and nuclear fractions. U6 and GAPDH were used as nuclear and cytoplasmic controls, respectively. Data are presented as mean ± SD from three independent experiments.

## Data Availability

The datasets used and/or analysed during the current study are available from the corresponding author on reasonable request.
