# Supplementary figures and images for: LncRNA HOXA11-AS promotes idiopathic pulmonary fibrosis progression via sponging miR-148a-3p and regulating SMAD2
Source: Hereditas. 2026 May 14;163:80. doi: 10.1186/s41065-026-00684-9 (PMC13343633; doi:10.1186/s41065-026-00684-9)

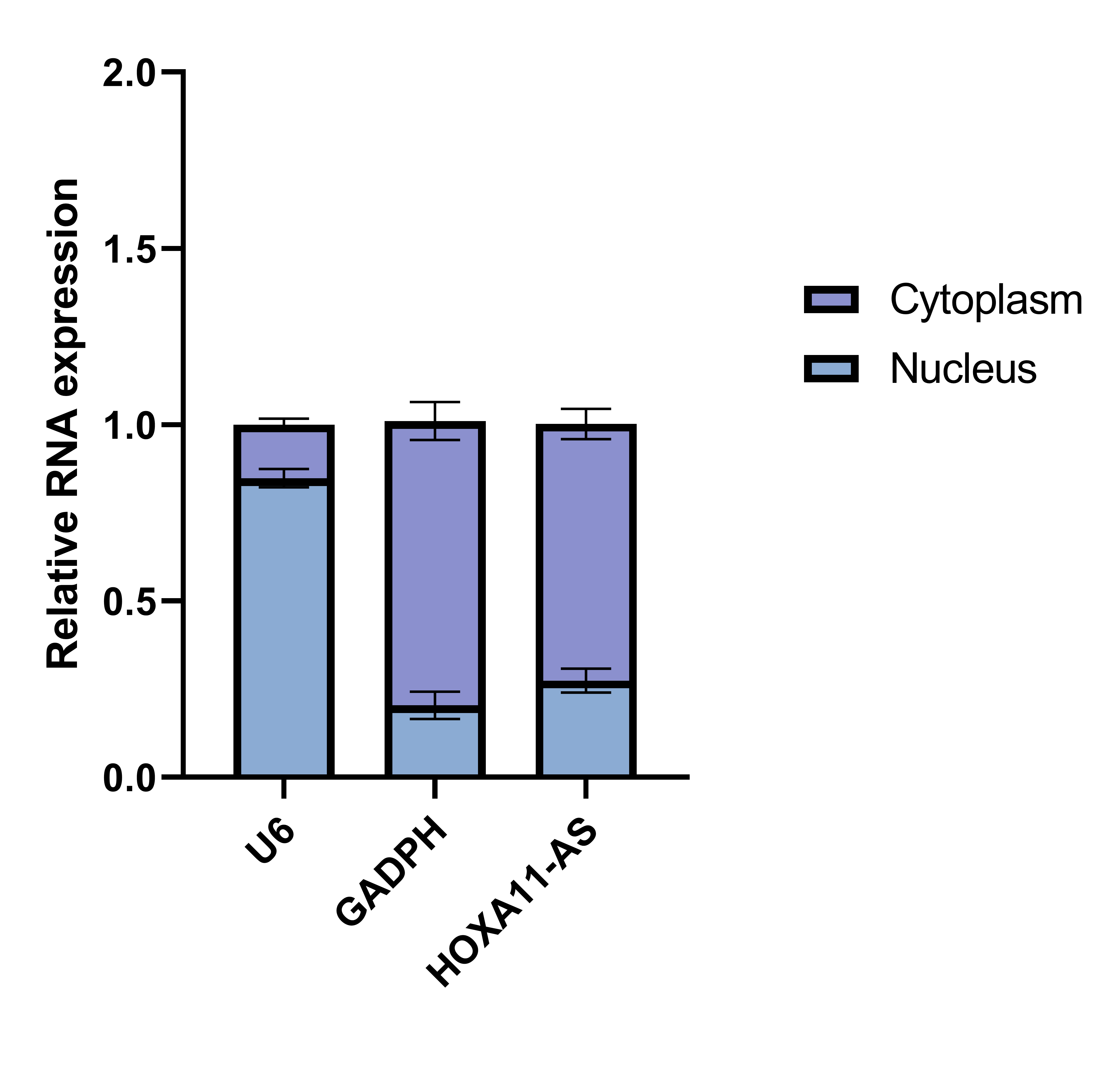

Supplement: Supplementary file 1 — Supplementary Material 1: Figure 1. Subcellular localization of HOXA11-AS in MRC-5 cells. Subcellular fractionation was performed in MRC-5 cells, followed by RT-qPCR analysis of HOXA11-AS expression in the cytoplasmic and nuclear fractions. U6 and GAPDH were used as nuclear and cytoplasmic controls, respectively. Data are presented as mean ± SD from three independent experiments. [file 41065_2026_684_MOESM1_ESM.tif]
